# Structure and Physiological Regulation of AMPK

**DOI:** 10.3390/ijms19113534

**Published:** 2018-11-09

**Authors:** Yan Yan, X. Edward Zhou, H. Eric Xu, Karsten Melcher

**Affiliations:** 1 Center for Cancer and Cell Biology, Van Andel Research Institute, 333 Bostwick Ave. N.E., Grand Rapids, MI 49503, USA; yan.yan@vai.org (Y.Y.); edward.zhou@vai.org (X.E.Z.); eric.xu@vai.org (H.E.X.); 2 VARI/SIMM Center, Center for Structure and Function of Drug Targets, CAS-Key Laboratory of Receptor Research, Shanghai Institute of Materia Medica, Chinese Academy of Sciences, Shanghai 201203, China

**Keywords:** energy metabolism, AMPK, activation loop, AID, α-linker, β-linker, CBS, LKB1, CaMKK2, αRIM

## Abstract

Adenosine monophosphate (AMP)-activated protein kinase (AMPK) is a heterotrimeric αβγ complex that functions as a central regulator of energy homeostasis. Energy stress manifests as a drop in the ratio of adenosine triphosphate (ATP) to AMP/ADP, which activates AMPK’s kinase activity, allowing it to upregulate ATP-generating catabolic pathways and to reduce energy-consuming catabolic pathways and cellular programs. AMPK senses the cellular energy state by competitive binding of the three adenine nucleotides AMP, ADP, and ATP to three sites in its γ subunit, each, which in turn modulates the activity of AMPK’s kinase domain in its α subunit. Our current understanding of adenine nucleotide binding and the mechanisms by which differential adenine nucleotide occupancies activate or inhibit AMPK activity has been largely informed by crystal structures of AMPK in different activity states. Here we provide an overview of AMPK structures, and how these structures, in combination with biochemical, biophysical, and mutational analyses provide insights into the mechanisms of adenine nucleotide binding and AMPK activity modulation.

## 1. AMPK Is a Master Regulator of Energy Homeostasis That Is Dysregulated in Disease

AMPK is the primary energy sensor and regulator of energy homeostasis in eukaryotes. It is activated by energy stress in response to increased ATP consumption (e.g., exercise, cell proliferation, anabolism) or decreased ATP production (e.g., low glucose levels, oxidative stress, hypoxia), which are sensed as low ratios of ATP to AMP and ADP. Upon activation, AMPK phosphorylates downstream targets to directly or indirectly modulate the activities of rate-limiting metabolic enzymes, transcription and translation factors, proliferation and growth pathways, and epigenetic regulators. Collectively, this increases oxidative phosphorylation, autophagy, and uptake and metabolism of glucose and fatty acids, and decreases the synthesis of fatty acids, cholesterol, proteins, and ribosomal RNAs (rRNAs), as well as decreasing cell growth and proliferation [1,2,3,4,5,6]. Due to its central roles in metabolism, AMPK is dysregulated in diabetes, obesity, cardiometabolic disease, and cancer, and it is a promising pharmacological target [1,2,5,7,8,9,10], especially for the treatment of type 2 diabetes [11,12,13].

## 2. AMPK Consists of a Stable Core Attached to Moveable Domains

**Figure 1:**
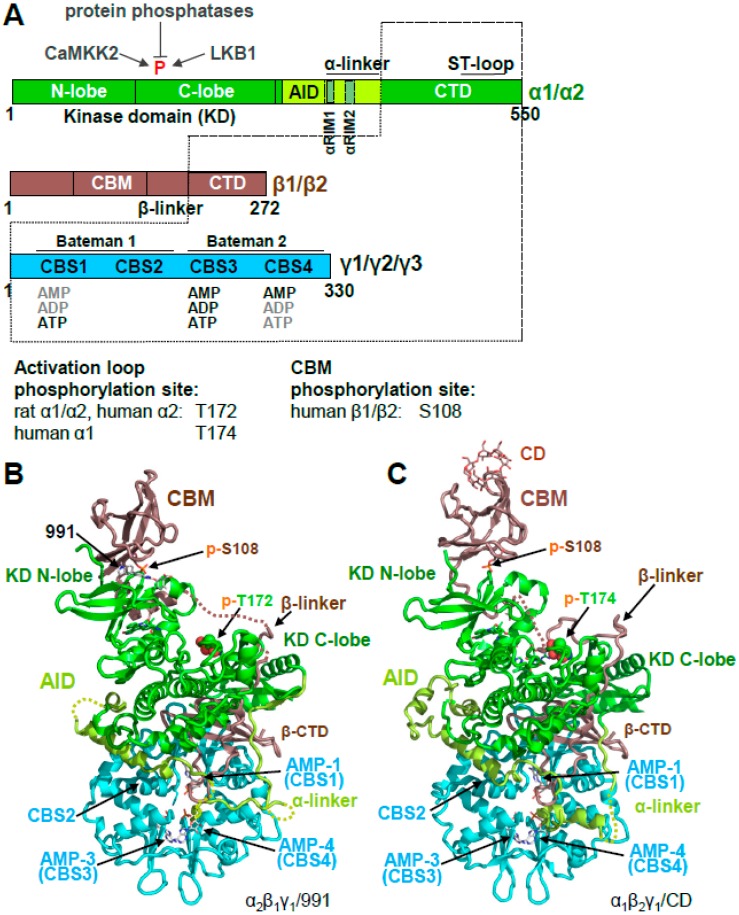
Overall structure of human adenosine monophosphate (AMP)-activated protein kinase (AMPK). (**A**). Domain structure and AMPK isoforms. Activation loop and carbohydrate-binding module (CBM) phosphorylation sites of different isoforms are indicated below the domain map (**B**,**C**). Crystal structures of phosphorylated, AMP-bound AMPK α_2_β_1_γ_1_/991 ((**B**); PDB: 4CFE) and α_1_β_2_γ_1_/cyclodextrin (CD) ((**C**); PDB: 4RER).

AMPK is a heterotrimeric αβγ protein kinase. In mammals, it is encoded by two alternative α subunits (α1 and α2), two alternative β subunits (β1 and β2), and three alternative γ subunits (γ1, γ2, and γ3) that can form up to 12 different αβγ isoforms [14]. The α subunits contain a canonical Ser/Thr kinase domain (KD), an autoinhibitory domain (AID), an adenine nucleotide sensor segment termed an α-linker, and a β subunit-interacting C-terminal domain (α-CTD), the latter of which contains the ST loop, which harbors proposed phosphorylation sites for AKT [15], PKA [16], and GSK [17]. The β subunits are composed of a myristoylated, unstructured N-terminus, a glycogen-binding carbohydrate-binding module (CBM), a scaffolding *C*-terminal domain (β-CTD) that interacts with both the γ subunit, and the α-CTD, and the extended β-linker loop that connects the CBM with the β-CTD (Figure 1A,B). The three alternative γ subunits consist of N-termini of different lengths and unknown function, followed by a conserved adenine nucleotide-binding domain that contains four cystathione β-synthetase (CBS) AMP/ADP/ATP binding sites (Figure 1). CBS1, 3, and 4 are functional, whereas in CBS2, the ribose-binding Asp residue is replaced by an Arg, and no nucleotide binding has been observed for CBS2 in heterotrimer structures.

**Figure 2:**
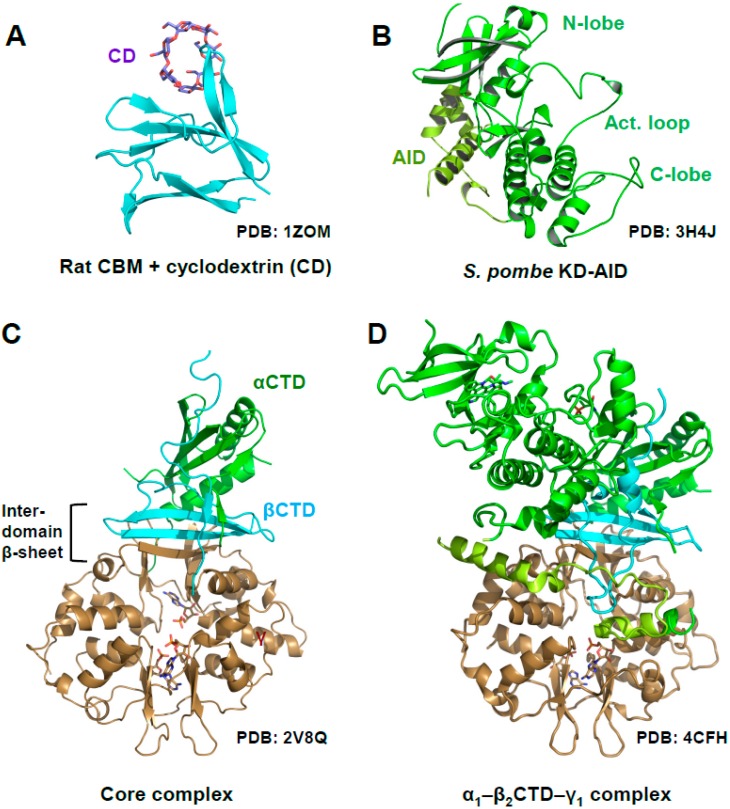
Structure of AMPK domains and subcomplexes. (**A**) Rat CBM bound to cyclodextrin; (**B**) Fission yeast kinase domain–autoinhibitory domain (KD‒AID) complex; (**C**) AMP-bound, phosphorylated mammalian AMPK core complex (rat α_1_-human β_2_-rat γ_1_); (**D**) AMP-bound, phosphorylated rat α_1_—human β_2_CTD—rat γ_1_ complex.

AMPK is a highly dynamic complex with a stable core formed by the γ subunit and the α- and β-CTDs, in which the β-CTD is sandwiched between the α and γ subunits (Figure 1A, core highlighted by dotted lines). Attached to the core are moveable domains whose position is determined by ligand binding and posttranslational modifications. As such, the holo-complex cannot be crystallized in the absence of multiple stabilizing ligands and/or protein engineering. Consequently, the first structures of AMPK consisted of isolated domains, e.g., the KD [18,19,20,21], the CBM bound to the glycogen mimic cyclodextrin [22], the yeast and mammalian nucleotide-bound scaffolding cores [23,24,25,26], the AID [27], and the yeast KD–AID complex [21] (Figure 2). 

### Activation Loop Phosphorylation Orchestrates the Catalytic Center for Phosphoryl Transfer

**Figure 3:**
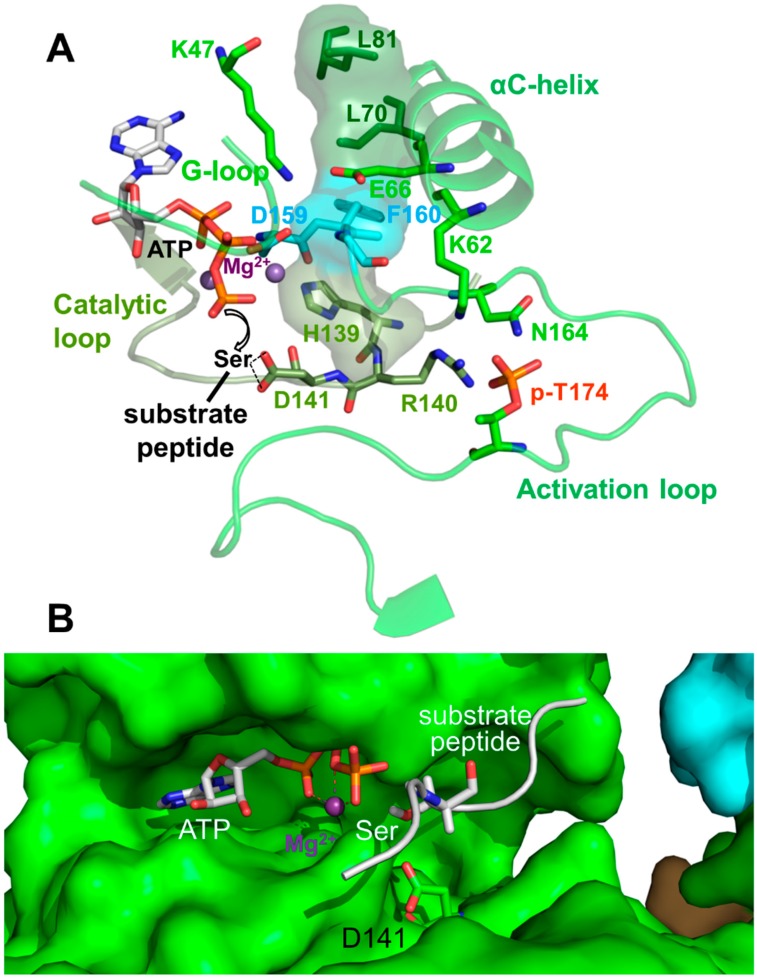
Active protein kinase catalytic cleft. (**A**) Key residues and structural elements of phosphorylated AMP-bound α_1_β_2_γ_1_ AMPK (4RER). Active kinase structures are characterized by a precisely positioned set of motifs for substrate- and adenosine triphosphate (ATP)-binding, in which four residues (L70, L81, H139, F160; shown in stick plus translucent surface presentation) are stacked against each other to form a regulatory spine. In this conformation, the activation loop p-T174 (p-T172 in human α_2_) positions R140 and D141 from the catalytic loop for peptide substrate binding, and K62 from the αC-helix for aligning the ATP-binding K47 and the Mg^2+^-binding DFG loop. The AMPK active protein kinase cleft resembles the canonical protein kinase A (PKA) site. To better visualize the active structure, we modeled the serine residue of a substrate peptide and the co-substrate ATP from the structure of PKA (PDB: 1ATP) in the catalytic cleft. Spheres: Mg^2+^ ions. (**B**) Surface presentation of the AMPK catalytic cleft (4RER) overlaid with a stick model of the aligned substrate peptide and ATP from the structure of substrate-bound CDK2 (PDB: 1QMZ). The Ser hydroxyl-positioning AMPK D141 is shown in green stick representation.

Kinase domains have a highly conserved structure consisting of a smaller *N*-terminal lobe (*N*-lobe), composed of a β-sheet and the αB and αC helices, and a larger α-helical *C*-terminal lobe (*C*-lobe; see Figure 1B and Figure 2B). The cleft between the lobes is the binding site for substrate peptides and Mg^2+^–ATP. The two lobes are separated by a flexible hinge at the back that allows them to move towards each other to cycle through substrate-accessible open and catalytically-competent closed conformations as part of the kinase catalytic cycle. Key regulatory elements of the KD are: (i) the activation loop at the entrance of the catalytic cleft; (ii) the αC helix in the N-lobe, which positions the ATP-binding lysine (K47 in human α1) and the Mg^2+^-binding DFG (Asp-Phe-Gly) loop; and (iii) the peptide substrate-binding catalytic loop in the C-lobe (Figure 3) [28,29,30]. 

AMPK belongs to the RD (Arg-Asp) kinases, in many of which phosphorylation stabilizes the activation loop through a charge interaction between the negatively charged activation loop phosphate, and the positively charged residues from the αC helix (K62 in AMPK α1), the activation loop (N164), and the catalytic loop (R140). This conformation in turn stabilizes the αC helix and positions the arginine (R) and adjacent aspartate (D) of the catalytic loop for substrate binding (Figure 3). The hallmark of active protein kinases is therefore a precisely positioned set of motifs for substrate- and ATP-binding, in which four residues from the catalytic loop (H139), the Mg^2+^-binding DFG loop (F160), the αC helix (L70) and the αC-αD loop (L81) are stacked against each other [28,29,30], as found in structures of active AMPK (Figure 3).

## 3. AMPK Is Activated Both by Direct Allosteric Activation and by Increasing Net Activation Loop Phosphorylation

AMPK activity is regulated at three different levels: at the level of (i) activation loop phosphorylation by upstream kinases, (ii) protection against activation loop dephosphorylation by protein phosphatases, and (iii) at the level of phosphorylation-independent, allosteric kinase activation (Figure 1A). Activation loop phosphorylation increases the AMPK activity by about 100-fold, while allosteric regulation changes AMPK activity up to ten-fold in mammalian cells and about two-fold in recombinant, bacterially produced AMPK [24,31,32,33]. AMP activates, and ATP inhibits, AMPK through all three mechanisms. ADP more weakly protects against activation loop dephosphorylation, does not allosterically activate AMPK [33,34,35,36], and it may not stimulate activation loop phosphorylation [33,37], although the latter is controversial [36].

The two main mammalian AMPK activation loop-phosphorylating kinases are the tumor suppressor LKB1 in complex with STRAD and MO25, and Ca^2+^/calmodulin-dependent protein kinase kinase β (CaMKK2) [38,39,40,41,42]. While CaMKK2 mediates Ca^2+^-dependent AMPK phosphorylation, AMP binding to the γ subunit increases activation loop phosphorylation through LKB1 by inducing a conformation that stabilizes formation of a complex between myristoylated AMPK, Axin, and LKB1/STRAD/MO25 [37,43]. However, the structural details of this interaction remain unknown. In addition, activation loop phosphorylation is also modulated by phosphorylation of the ST loop [15,16,17] and by ubiquitination of AMPK [44] and LKB1 [45]. 

In addition to adenine nucleotides, glucose, glycogen, and nicotinamide adenine dinucleotides are also important energy metabolites. Glucose has recently been identified as an important AMPK activity regulator, but it does so without direct AMPK binding [43,46]. In contrast, both glycogen and NADPH and NADH can directly bind AMPK: glycogen at the CBM [22,47], and in a reconstituted system, NADPH and NADH at the adenine nucleotide sensor site CBS3 [34,48]. However, the physiological relevance of the glycogen [47,49,50] and NADPH/NADH [34,48] interactions for AMPK activity regulation remains unclear.

Finally, a number of pharmacological activators bind AMPK at a unique site at the interface between CBM and KD (so called allosteric drug and metabolite [ADaM] site), as first shown for Merck compound 991 [51], and derivatives of the Abbot compound A769662 [52]. Binding greatly stabilizes the association of the highly dynamic CBM with the KD [53], an interaction that is also modulated by CBM phosphorylation and carbohydrate binding [49,53]. ADaM site agonists activate AMPK both directly and through increased protection against activation loop dephosphorylation, whose structural details will be covered in detail in a separate article in this issue.

Besides activity regulation, the level of AMPK is regulated by ubiquitination and proteasomal degradation in brown adipose tissue [54], testis [55], certain cancers [55,56], and in the presence of high levels of glucose [57]. 

### 3.1. The γ Subunit Contains Three Functional Adenine Nucleotide Binding Sites

**Figure 4:**
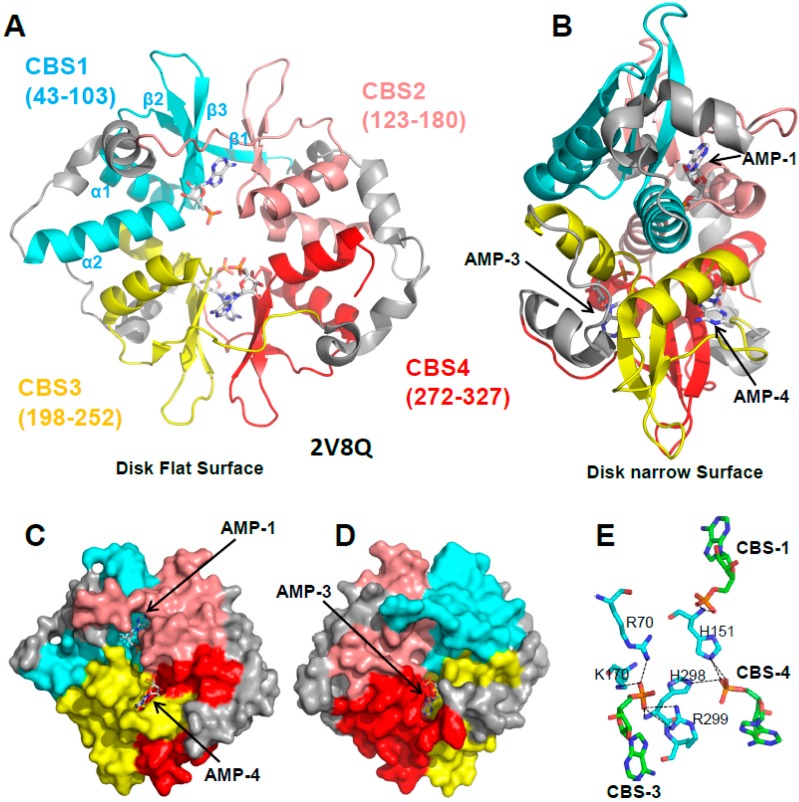
AMP binds three of the four CBS sites of the γ subunit. (**A**,**B**) Cartoon representation of the γ subunit in two different orientations. AMP molecules are shown in stick representation. The four CBS sites are shown in different colors with the secondary structure elements of CBS1 labeled. (**C**,**D**) Surface representation of the front and back sides of the disk flat surfaces illustrating the AMP-occupied binding pockets 1, 3, and 4, and the empty CBS2 pocket. (**E**) The phosphate groups (orange) of the three AMP molecules (cyan C atoms) coordinately interact with a set of polar γ subunit residues (green C atoms); O: red, N: blue.

The structure of the yeast and mammalian AMPK core scaffolds revealed a disk-shaped γ subunit composed of four CBS sites. Each CBS consists of a strand-helix-strand-strand-helix fold (β1-α1-β2-β3-α2) with long intervening loops (Figure 4A). β1 is often incomplete, but where present, it forms a three-stranded sheet with the two central β-strands (β2 and β3). The β-sheet of one CBS packs parallel with the sheet of a neighboring CBS. The interface between the two sheets forms two clefts, one on the top flat side and one on the bottom flat side of the disk, which are the binding sites for adenine nucleotides (Figure 4C,D). Therefore, each binding site requires a tandem CBS pair to form a functional unit termed the Bateman domain (CBS1 + CBS2 = Bateman domain 1, CBS3 + CBS4 = Bateman domain 2). The structures of the core complexes in the presence of AMP [24,58], ADP [34], or ATP [24,58] revealed adenine nucleotide binding at three sites in mammalian AMPK: CBS1, CBS3, and CBS4. 

### 3.2. CBS3 Is the Adenine Nucleotide Sensor Site

While the structure of the core complexes revealed how adenine nucleotides bind the γ subunit, they did not provide information on how the binding signal is transduced to the KD in the α subunit. In 2011, the Gamblin and Carling groups crystallized an AMPK complex containing rat α1, human β2 CTD, and rat γ1 [34]. While this complex is not regulated by protection against activation loop dephosphorylation [51], it retained direct AMPK activation by AMP and ADP. The structure revealed that the α-linker that connects AID and α-CTD directly bound the γ subunit [34], which has been validated in all subsequent AMP-bound AMPK complex structures with a resolved α-linker. A segment of the linker, termed regulatory subunit-interacting motif 2 (αRIM2) [27,59], interacts with AMP at CBS3, suggesting that αRIM2 functions as an adenine nucleotide sensor, and that it mediates the transduction of the adenine-binding signal to the KD [27,34,59]. This function has been validated by several experimental approaches. First, the mutation of either of the two key αRIM2 residues (E362 and R363 in rat α1 and human α2; E364 and R365 in human α1) abolished or largely reduced both AMP-dependent direct AMPK activation [27,49,51] and AMP-dependent protection against activation loop dephosphorylation [49]. Second, AMP increases, and ATP decreases the interaction between isolated α-linker and core AMPK in a reconstituted system, and the AMP increase requires intact E364 and R365 [49]. Third, the AMP-mimetic synthetic AMPK activator C2 activates AMPK α-isotype-selectively (it fully activates α1-containing complexes, but only partially α2 complexes), and this selectivity can be fully reversed by a swap of the αRIM2 regions [60,61].

## 4. If CBS3 Is the Sensor Site, What Are the Roles of CBS1 and CBS4?

Of the three functional CBS sites, only CBS3 interacts with the α subunit, an interaction that is directly modulated by AMP and ATP. In contrast, CBS1 and CBS4 do not interact with any part of the α- or β-subunit. Moreover, CBS4 binds AMP very tightly [24,58] and it is unlikely to exchange AMP under physiological conditions, yet mutations in CBS4 abolish regulation by AMP [36,58], while mutations in CBS1 have either no [58] or only a small [36] effect on AMPK regulation. Important insight came from a mutational study. When CBS1, CBS4, and the ATP-binding site in the KD are mutated, so that CBS3 remains the only functional adenine nucleotide binding site, it binds AMP only very weakly and with 10–100 times lower affinity than ATP [48]. Since the cellular ATP concentrations are much higher than AMP and ADP concentrations, CBS3 by itself would remain almost completely ATP-bound under both normal and energy stress conditions. However, the phosphates of adenine nucleotides bound to CBS1, 3, and 4 coordinately bind a set of charged and polar amino acids (Figure 4E), so that binding to one site affects binding to the other two sites. Through these coordinated interactions, AMP bound at CBS4, together with additional interactions from αRIM2, stabilizes AMP at CBS3. This increases CBS3’s affinity for AMP by two orders of magnitude, and its AMP/ATP binding preference by two to three orders of magnitude [48], allowing CBS3 to sensitively detect physiological energy stress versus non-stress adenine nucleotide levels. Conversely, both CBS3 and CBS1 strongly stabilize AMP-binding at CBS4, so that under physiological conditions CBS4 remains essentially non-exchangeably AMP-bound and CBS1 largely ATP-bound [48].

## 5. AMP-Binding at CBS3 Destabilizes an Inhibitory AID–KD Interaction

**Figure 5:**
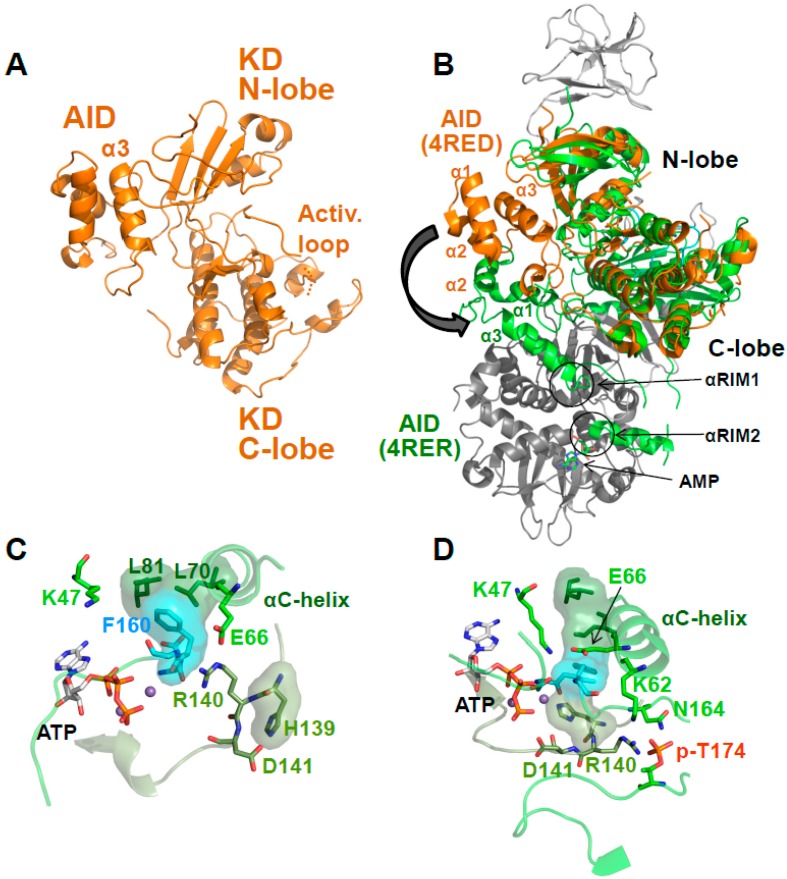
The AID is in equilibrium between KD- and γ-bound conformations. (**A**) Cartoon structure of the human α_1_ KD-AID complex. (**B**) Overlay of the inactive KD-AID structure with the structure of active holo-AMPK (α subunit: green; β- and γ-subunits: grey). The arrow indicates the repositioning of the AID in the active structure. (**C**,**D**) Catalytic center of the inactive (**C**) and active (**D**) AMPK conformation. Stick plus translucent surface presentations indicate the regulatory spine residues L70, L81, H139, and F160. Mg^2+^-ATP was modeled into both structures for orientation, even though it cannot bind to the inactive structure shown in panel C. Spheres: Mg^2+^ ions.

The KD is followed by the AID, a small 48 amino acid domain that inhibits kinase activity about tenfold in the context of a KD–AID fragment [62,63]. Crystal structures of the fission yeast [21] and human [49] AMPK KD–AID fragments revealed a three-helical AID, whose *C*-terminal helix (α3) directly binds the hinge between the KD *N*- and *C*-lobes at the backside of the KD (Figure 2B and Figure 5A). In contrast, in structures of active, AMP-bound AMPK [34,49], the AID is rotated away from the KD and bound to the γ subunit (see structure overlay in Figure 5B). The AID–KD interaction arrests the KD in a unique inactive conformation, in which the ATP binding K47, the Mg^2+^-binding DFG loop, and the substrate-binding catalytic loop are misaligned, and H139 of the regulatory spine is out of register [21,49] (so called “HRD-out” conformation [30]; Figure 5C). The inhibitory function of the KD–AID interaction was further validated by the mutation of interface residues in either the KD or the AID, all of which made AMPK constitutively active [21,49]. Conversely, binding of the AID to the γ subunit, as seen in structures of AMP-bound AMPK [34,49], allows the KD to adopt the active conformation [27,34,51,59] (Figure 5D). Consistently, mutations in AID-interacting γ subunit residues make AMPK constitutively inactive [59]. 

### A Highly Conserved Interaction Network Links αRIM2/CBS3 and AID-αRIM1/CBS2 Binding

**Figure 6:**
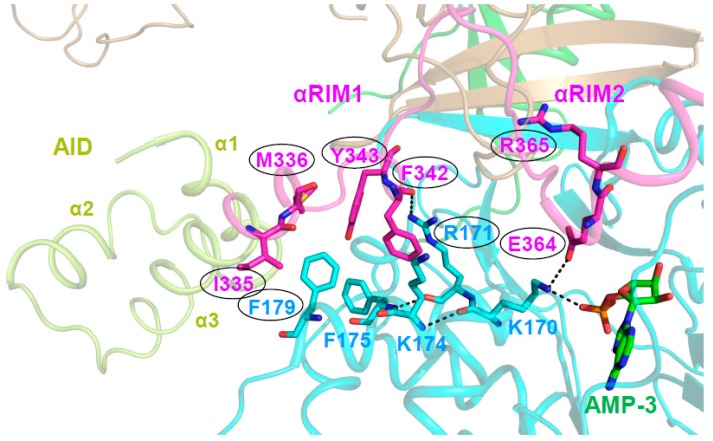
αRIM2/CBS3 and AID-αRIM1/CBS2 interactions are linked. Structure of human AMP-bound AMPK α_1_β_2_γ_1_ (4RER) with key residues shown in a stick presentation; the α-linker is shown in magenta, the γ subunit in cyan, and the AID in light green. AMP bound at CBS3 and αRIM2 E364 directly interact with γ1 K170, which positions the αRIM1-binding residues R171, and indirectly through K174 and F175, F179, thus stabilizing the AID‒γ subunit interaction. Consistently, mutations of the αRIM1/γ subunit (and αRIM2/CBS3) interface residues highlighted by oval outlines (human α_1_: F342D/Y343D, I335D/M336D, E364, R365; γ_1_: R171A, F179D) are constitutively AMP-non-responsive. Dashed lines indicate hydrogen bonds.

The structure of AMP-bound AMPK α_1_‒β_2_CTD‒γ_1_ [34] first revealed the AID conformation in active AMPK, in which the border of the AID and the N-terminus of the α-linker, termed αRIM1, binds the γ-subunit at the unoccupied CBS2 [27,34,51,59]. Mutational analysis by Ja-Wei Wu’s group provided a molecular pathway to link αRIM2 binding of AMP-occupied CBS3 to direct AMPK kinase activation. They first showed that αRIM1/CBS2 interface amino acids corresponding to human α1 I335/M3364 and F342/Y343, and human γ1 R171 and F179 are required for AMP-mediated relief of AMPK autoinhibition [59]. In active, AMP-bound holo-AMPK, the direct interaction of γ1 K170 with both AMP/CBS3 and αRIM2 α1 E364 positions three key residues at the αRIM1 interface. First, the residue following K170, R171, forms Van der Waals interactions and a backbone hydrogen bond with αRIM1 α1 F342. Second, the K170-interacting residues K174 and F175 form Van der Waals bonds with F342 and both Van der Waals and π-stacking interactions with γ1 F179. The latter is the linchpin of the interface and directly interacts with all four αRIM1 residues that are required for the relief of AMPK autoinhibition (I335, M336, Y343, and F342; Figure 6). Similarly, E364, R171, and F179 are also all required for the relief of AMPK autoinhibition [59]. The mutational analysis thus provides strong support that this AMP-stabilized interaction network that is seen in all active structures of holo-AMPK is responsible for shifting the AID equilibrium from the inactive, KD-bound conformation to the active, γ/CBS2-bound conformation.

ATP binding is thought to disrupt this network. In the structure of the core AMPK complex co-crystallized with ATP [58], ATP was bound to CBS4 and CBS1, which sterically interfered with nucleotide binding at CBS3, and caused rearrangement and disruption of the interaction network [58,59]. However, the physiological relevance of this structure remains unclear, since under physiological conditions, CBS4 does not seem to exchange AMP (see above; [24,48,58]). Therefore, a final understanding of how ATP disrupts the CBS3–α-linker–AID network will require the structure of the holo-AMPK complex, including the α-linker, in ATP-bound conformation.

ADaM site ligands, while not focus of this review, directly activate AMPK by a completely different mechanism. Through binding of both the CBM and the KD [51,52] and stabilization of the CBM–KD interaction [53], the *N*-terminus of the β-linker at the CBM border adopts a helix that packs parallel to the αC-helix, and it has therefore been named *C*-interacting helix [51]. This suggested that ADaM site ligands may activate AMPK by stabilizing αC through induced formation of the *C*-interacting helix, reminiscent of the regulatable αC stabilization of several other protein kinases [64]. Support for this model came from the mutation of H233 in the *C*-interacting helix, which reduced activation by the ADaM site ligand 991 [51], and by direct demonstration through hydrogen/deuterium exchange mass spectrometry (HDX-MS) that 991 binding strongly and selectively stabilizes αC [48].

## 6. Regulation of Activation Loop Accessibility

**Figure 7:**
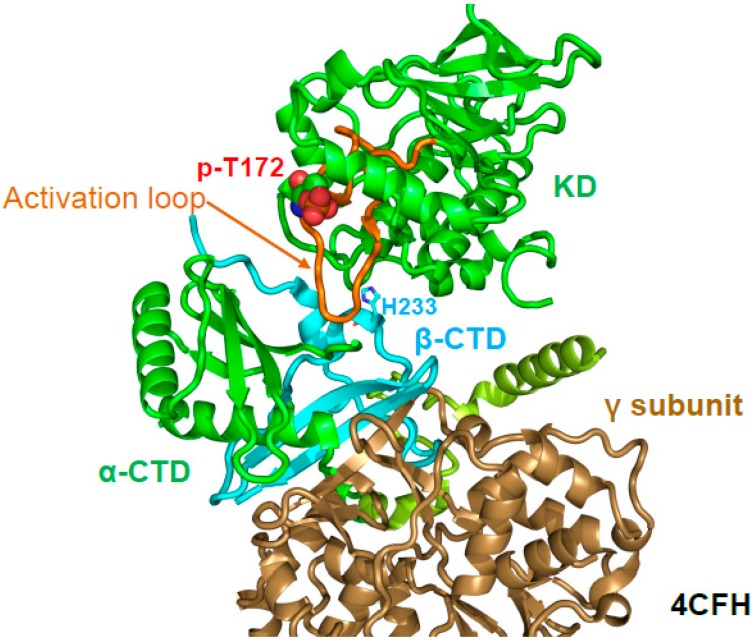
The β-CTD binds and stabilizes the activation loop. Structure of AMP-bound, phosphorylated AMPK α_1_–β_2_CTD–γ_1_ (PDB: 4CFH). The activation loop is highlighted in orange, and p-T172 is shown in sphere presentation.

A major regulatory mechanism for AMPK activation by AMP and ADP is the protection of activation loop p-T172 (human α1 T174) against dephosphorylation. p-T172 protection can be demonstrated in a cell-free, reconstituted system independent of the phosphatase used (e.g., PP2C, PP2A, λ-phosphatase), and AMP does not, or only slightly inhibit the dephosphorylation of a different substrate, casein, by PP2Cα [32]. Therefore, reduced dephosphorylation is not due to phosphatase inhibition, but to an AMP/ADP-induced change in the activation loop accessibility. The crystal structure of AMP-bound, phosphorylated AMPK α_1_–β_2_CTD–γ_1_ (PDB: 4CFH) first demonstrated that the activation loop directly interacts with the core of AMPK [34]. Specifically, the stable β-CTD directly bound and stabilized the activation loop (Figure 7). The authors therefore proposed that the core shields the activation loop from phosphatase access. In agreement, mutation of the activation loop-interacting β2 H235 increased p-T172 dephosphorylation in the context of holo-AMPK [34]. However, the construct used in the structure was not regulated by protection against activation loop dephosphorylation [51], indicating that additional parts of AMPK, likely either the β-linker and/or the CBM, were also required for AMP-mediated, and probably ADaM site ligand-mediated protection against activation loop dephosphorylation. Consistently, in structures in which the β-linker is largely resolved (e.g., β2-linker in 4RER [49], β1-linker in 5ISO [65]), p-T172 is clearly protected by the β-linker, especially in the case of the β2-linker. Finally, how can the activation loop in AMP-bound conformation be largely inaccessible to protein phosphatases without affecting accessibility to the T172-phosphorylating upstream protein kinases? Answers to these fundamental questions will likely require the structure of holo-AMPK in the alternative, ATP-bound state and analysis of AMPK’s conformational landscape and dynamics in solution. 

## 7. Conclusions and Future Directions

AMPK is a molecular machine consisting of the adenine nucleotide-binding core (γ subunit plus α- and β-CTDs), the catalytic KD, and at least four dynamic domains (AID, CBM, and the α- and β-linkers). We propose that adenine nucleotides, ADaM site ligands, and CBM phosphorylation affect the conformation of the KD through induced movements of the dynamic domains, while phosphorylation of activation loop and S/T loop modulate the KD conformation directly. Through concerted efforts, the mechanism of direct, allosteric AMPK activation through AID movement and αC stabilization is relatively well understood. However, the structural basis of direct inhibition by ATP, of activation loop accessibility regulation through ligands and possibly phosphorylation, and of the AMP-induced interaction with Axin and the LKB1 complex all remain poorly understood. The most important future challenges in AMPK structural biology will therefore be the determination of the structures of holo-AMPK in its inhibited, ATP-bound conformation, and in complex with Axin and LKB/STRAD/MO25.

## Abbreviations

ADaM site	Allosteric drug and metabolite-binding site
AID	Autoinhibitory domain
AMPK	AMP-activated protein kinase
αRIM	α-regulatory subunit interaction motif
CaMKK2	Ca^2+^/calmodulin-dependent protein kinase kinase β
CBM	Carbohydrate-binding module
CBS	Cystathionine β-synthetase
CTD	C-terminal domain
HDX-MS	Hydrogen deuterium exchange mass spectrometry
KD	Kinase domain
LKB1	Liver kinase B1
MO25	Mouse protein-25
PP2A	Protein phosphatase 2A
PP2C	Protein phosphatase 2C
STRAD	STE20-related kinase adaptor
